# Cortisol-to-DHEAS Awakening Ratio Varies With Dementia Severity, Agitation, Age, and Sex

**DOI:** 10.1093/geroni/igaf122.3565

**Published:** 2025-12-31

**Authors:** Wanrui Wei, Töres Theorell, Gabriella Engstrom, Azita Emami

**Affiliations:** Yale University, Orange, Connecticut, United States; Karolinska Institutet, Stockholm, Stockholms Lan, Sweden; Florida Atlantic University, Boca Raton, Florida, United States; Yale University, Orange, Connecticut, United States

## Abstract

The cortisol-to-dehydroepiandrosterone sulfate (DHEAS) ratio in the awakening response has emerged as a promising biomarker of stress-related dysregulation in neurodegenerative conditions. However, little is known about how this ratio varies among people with dementia (PWD) and their family caregivers, particularly across demographic and clinical subgroups. This study aimed to compare awakening cortisol-to-DHEAS ratios between PWD and caregivers and to examine whether age, sex, agitation, and dementia severity predict hormonal imbalance using nonlinear modeling. We analyzed 1,093 saliva samples from 32 PWD and 31 family caregivers. Log-transformed cortisol-to-DHEAS ratios were evaluated through group comparisons, stratified regressions, and generalized additive models. No significant overall group difference emerged (p = 0.405). However, subgroup analyses revealed elevated ratios in males under 75 with dementia (p = 0.048, Cliff’s δ = 0.15). Nonlinear age effects were observed across subgroups, particularly among female caregivers and PWD. Dementia severity was positively associated with ratio elevation, while agitation showed a wave-like nonlinear pattern. In sex-stratified regressions, dementia severity, agitation, and quadratic age terms significantly predicted ratio variation in males (R² = 0.221) but not in females. These findings suggest that the awakening cortisol-to-DHEAS ratio reflects nonlinear, age- and sex-specific patterns of stress regulation. The ratio may serve as a sex-sensitive biomarker of neuropsychiatric vulnerability in dementia, highlighting the need for tailored approaches in neuroendocrine aging research.

